# First record of *Jerzego* Maddison, 2014, with description of a new species from China (Araneae, Salticidae)

**DOI:** 10.3897/BDJ.13.e156957

**Published:** 2025-06-16

**Authors:** Cheng Wang, Jiahui Gan, Xiaoqi Mi

**Affiliations:** 1 Guizhou Provincial Key Laboratory for Biodiversity Conservation and Utilization in the Fanjing Mountain Region, Tongren University, Tongren, China Guizhou Provincial Key Laboratory for Biodiversity Conservation and Utilization in the Fanjing Mountain Region, Tongren University Tongren China

**Keywords:** Hisponinae, morphology, salticid, south-western China, taxonomy

## Abstract

**Background:**

*Jerzego* Maddison, 2014, the only recorded Asian genus of the subfamily Hisponinae Simon, 1901, is represented by four tree bark dwellers restricted to Malaysia, India, Indonesia and Sri Lanka.

**New information:**

A new jumping spider, *Jerzegoqiuhangi* **sp. nov.** is described, based on both sexes from Xishuangbanna, Yunnan, China. Diagnostic photos of the habitus and copulatory organs and a distributional map are provided.

## Introduction

*Jerzego* Maddison, 2014 is characterised by the presence of a constriction between the small eyes and posterior eyes (Maddison and Piascik 2014). The genus was erected based on both the morphological and molecular evidence by Maddison and Piascik (2014), who also described a new species as the generotype and transferred two members from *Hispo* Simon, 1886. After that, only one new member was reported from India by Sanap et al. (2019) with both sexes. Although the genus is well-defined, further taxonomic attention is also necessary because one of the members is known from juveniles and another one is only known from single females (World Spider Catalog 2025).

In November 2024, four bark-dwelling jumping spiders were collected from Xishuangbanna, China. After being compared with congeners morphologically, they were recognised as a new species of the Chinese unrecorded genus *Jerzego*, which is described as *J.qiuhangi* **sp. nov** herein.

## Materials and methods

Specimens were collected by hand. They were preserved in 95% ethanol and are deposited at the Museum of Tongren University (TRU) in Tongren, China. Specimens examination and photo generation methods followed Wang et al. (2024).

All measurements are given in millimetres. Leg measurements are given as: total length (femur, patella, tibia, metatarsus, tarsus). Abbreviations used in the text and figures are including: **AERW** anterior eye row width; **AG** accessory gland; **ALE** anterior lateral eye; **AME** anterior median eye; **At** atrium; **CD** copulatory duct; **CO** copulatory opening; **E** embolus; **EFL** eye field length; **FD** fertilisation duct; **PERW** posterior eye row width; **PME** posterior median eye; **PLE** posterior lateral eye; **RTA** retrolateral tibial apophysis; **S** spermatheca; **SD** sperm duct.

## Taxon treatments

### Jerzego qiuhangi

Wang, Gan & Mi sp. nov.

190B677C-35DA-5C76-9ECD-9B7E349C185B

82E8656F-2F9F-496E-9A33-4170FFF08F50

#### Materials

- **Type status:** Holotype. **Occurrence:** recordedBy: Hang Qiu; individualID: TRU-JS 0849; sex: male; occurrenceID: 6181C211-20BB-55C7-8A5D-21794542EEEA; **Taxon:** scientificNameID: *Jerzegoqiuhangi* sp. nov.; **Location:** country: China; county: Menghai; locality: Mankuyao Village, around of Manyao Reservoir; verbatimElevation: unspecified; verbatimLatitude: 21°56.93'; verbatimLongitude: 100°28.27'; **Identification:** identifiedBy: Cheng Wang; **Event:** samplingProtocol: by hand; year: 2024; month: 11; day: 27; habitat: tree bark
- **Type status:** Paratype. **Occurrence:** recordedBy: Hang Qiu; individualID: TRU-JS 0850–0852; individualCount: 3; sex: 2 males, 1 female; occurrenceID: EAC5F56C-F8EB-5FFD-A7EA-AE6160963310; **Taxon:** scientificNameID: *Jerzegoqiuhangi* sp. nov.; **Location:** country: China; county: Menghai; locality: Mankuyao Village, around of Manyao Reservoir; verbatimElevation: unspecified; verbatimLatitude: 21°56.93'; verbatimLongitude: 100°28.27'; **Identification:** identifiedBy: Cheng Wang; **Event:** samplingProtocol: by hand; year: 2024; month: 11; day: 27; habitat: tree bark

#### Description

**Figure 1.**
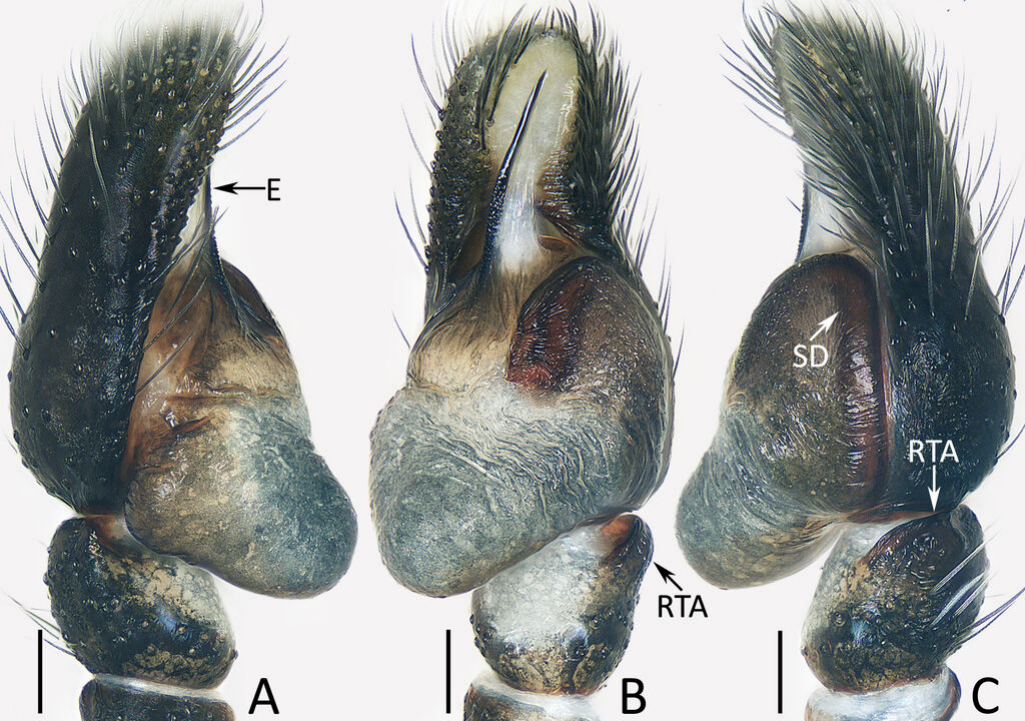
Male palp of *Jerzegoqiuhangi* sp. nov., holotype. **A** prolateral; **B** ventral; **C** retrolateral. Scale bars 0.1 mm.

Male (Fig. 1A–C, Fig. 3A, B and D–F). Total length 5.25. Carapace 2.44 long, 1.69 wide. Abdomen 2.75 long, 1.41 wide. Eye sizes and inter distances: AME 0.42, ALE 0.21, PLE 0.19, AERW 1.16, PERW 1.25, EFL 0.98. Legs: I 4.06 (1.38, 0.85, 0.80, 0.53, 0.50), II 3.41 (1.00, 0.70, 0.75, 0.48, 0.48), III 3.11 (0.95, 0.55, 0.60, 0.63, 0.38), IV 4.14 (1.20, 0.73, 0.98, 0.85, 0.38). Carapace dark, flat, bearing short white scales bilaterally and posteriorly; fovea longitudinal. Chelicerae each with three promarginal and retromarginal teeth. Endites elongated, with pale disto-inner areas. Labium almost as long as wide. Sternum about 1.5 times longer than wide, covered with dark thin setae and with rather pointed posterior end. Legs yellowish-brown to dark, covered with sparse white scales, with enlarged femora and tibiae Ⅰ and bearing dense, long setae ventrally on patellae and tibiae Ⅰ. Abdomen elongated, dorsum dark, covered with sparse white scales anteriorly, with sub-fusiform scutum on median line of anterior 1/3, followed by pair of dark grey, elongate-oval patches and pair of inclined white stripes of scales on posterolateral side; venter with four dotted lines extending from epigastric furrow to terminus.

Palp (Fig. 1A–C): tibia almost as long as wide in retrolateral view, with broad retrolateral apophysis slightly more than half its width; cymbium about 1.5 times longer than wide; bulb swollen medio-posteriorly, with well-developed posterior lobe extending towards postero-prolateral side; embolus straight, originating from anteroprolateral portion of bulb, with inner membranous portion and rather pointed tip.

**Figure 2.**
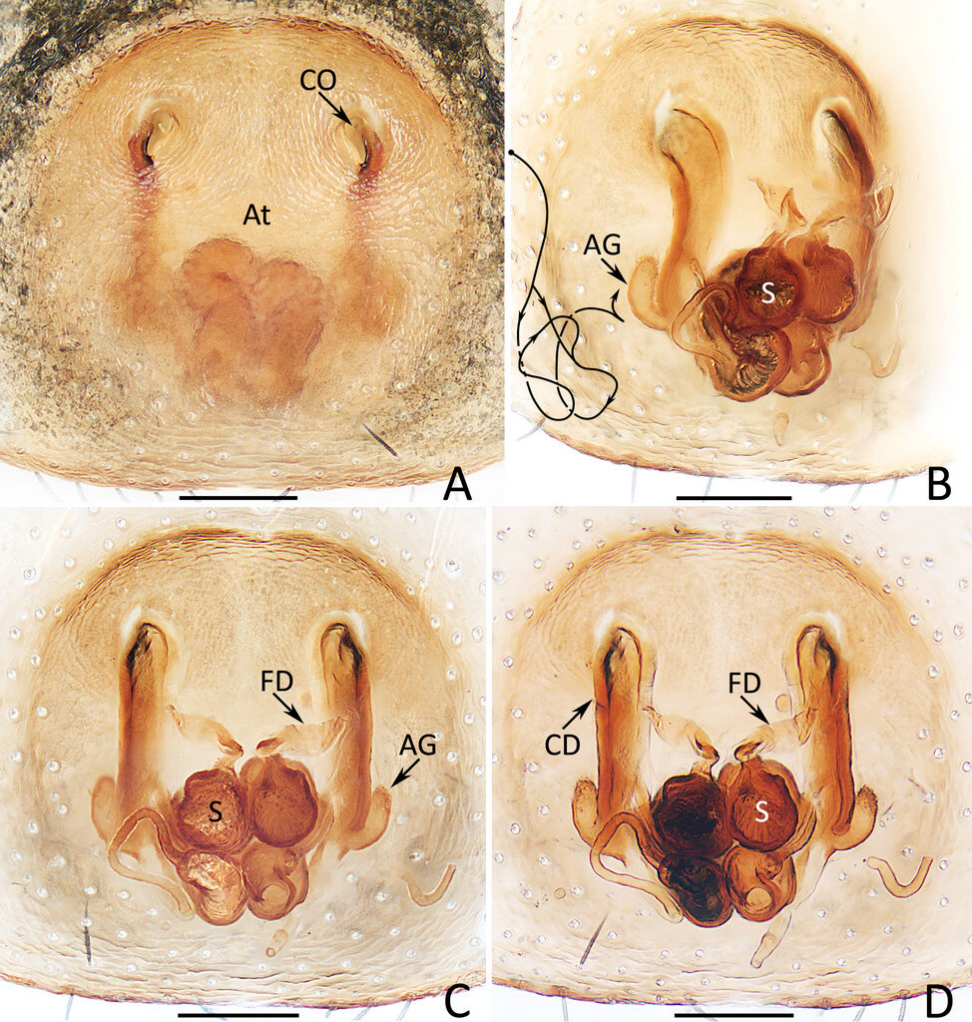
Epigyne of *Jerzegoqiuhangi* sp. nov. **A** epigyne, ventral; **B** vulva, dorsolateral; **C, D** ditto, dorsal. Scale bars 0.1 mm.

**Figure 3.**
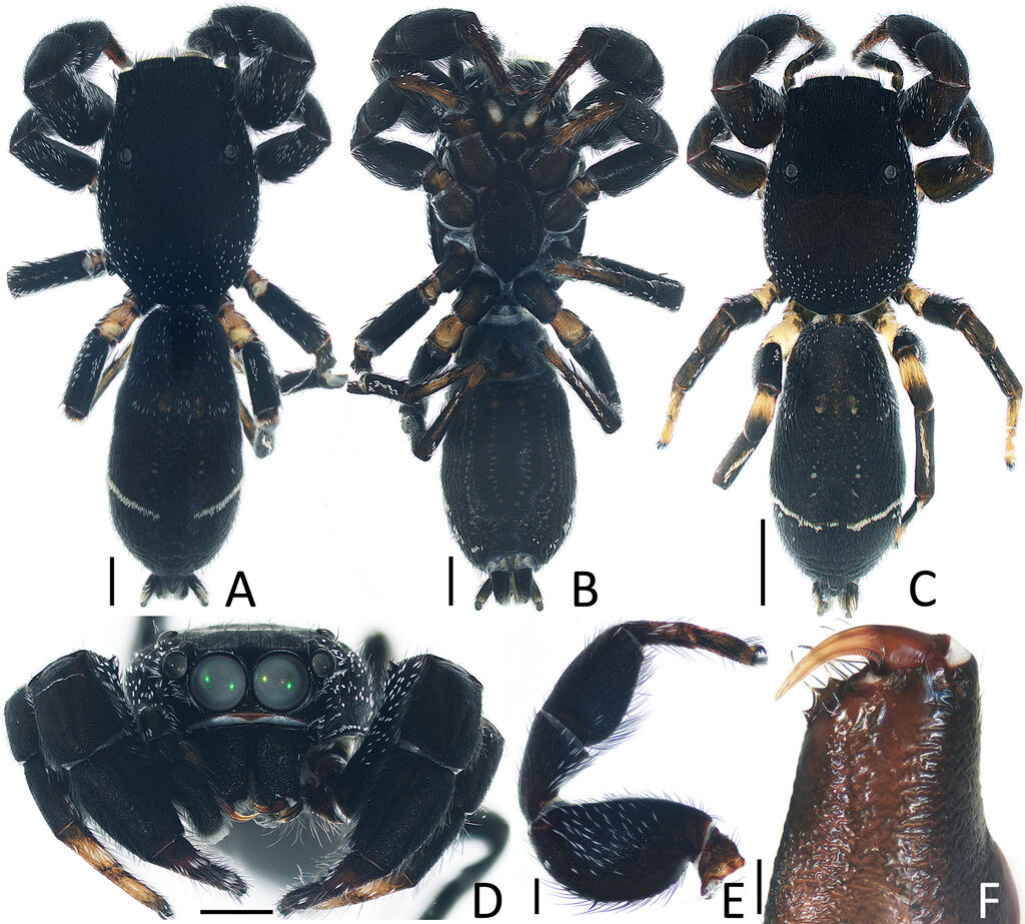
*Jerzegoqiuhangi* sp. nov. **A**, **B**, **D**–**F** (holotype); **C** (female paratype). **A**, **C** habitus, dorsal; **B** ditto, ventral; **D** carapace, frontal; **E** leg Ⅰ, prolateral; **F** chelicera, posterior. Scale bars **A**, **B**, **D**, **E** = 0.5 mm; **C** = 1.0 mm; **F** = 0.1 mm.

Female (Fig. 2A–D and Fig. 3C). Total length 5.93. Carapace 2.71 long, 1.82 wide. Abdomen 3.11 long 1.61 wide. Eye sizes and inter distances: AME 0.43, ALE 0.22, PLE 0.20, AERW 1.32, PERW 1.36, EFL 1.14. Legs: I 4.02 (1.33, 0.73, 0.88, 0.58, 0.50), II 3.68 (1.10, 0.75, 0.85, 0.53, 0.45), III 3.24 (0.98, 0.53, 0.65, 0.65, 0.43), IV 4.54 (1.33, 0.78, 1.05, 0.95, 0.43). Habitus similar to that of male, except without dorsal abdominal scutum.

Epigyne (Fig. 2A–D): almost as long as wide, with well-developed, oval atrium with arc-shaped anterior ridge; copulatory openings elongate-oval, separated from each other by about 2/3 atrial width; copulatory ducts posteriorly extending with slight curves at proximal, medio-proximally divided into much thinner, straight, bar-shaped dorsal branch and irregular curved ventral branch and both branches fused at position of posterior 1/3, remainder portions broadened and curved into nearly C-shape in lateral view; accessory glands mediolaterally located on proximal 1/3 of copulatory ducts, curved about 70° at beginning and then running anteriorly; spermathecae nearly spherical, touching each other; fertilisation ducts originating from anterior portions of spermathecae.

#### Diagnosis

The male of *Jerzegoqiuhangi* sp. nov. resembles that of *J.corticicola* Maddison 2014 in the general shape of the palp, but can be easily distinguished by the embolus, which is about half the bulb length and has an inner membranous portion (Fig. 1B) vs. about 1/4 the bulb length and lacks similar membranous portion in *J.corticicola* (Maddison and Piascik (2014): fig. 2). The female of this new species resembles that of *J.bipartitus* (Simon, 1903) in having the similar habitus pattern and epigyne, but can be easily distinguished by the lack of epigynal septum (Fig. 2A) vs. present in *J.bipartitus* (Wanless (1981): fig. 12F). The species also somewhat resembles that of *J.sunillimaye* Sanap, Caleb & Joglekar, 2019 in having rather consistent dorsal patterns of the abdomen, but differs by the straight embolus originating from the anteroporlateral portion of tegulum and has a pointed tip and by the lack of median septum (Fig. 1B and Fig. 2A) vs. twisted embolus originating from the central portion of tegulum and has a blunt tip and the presence of median septum in *J.sunillimaye* (Sanap et al. (2019): figs 5, 7, 9, 16 and 18).

#### Etymology

The specific name is a patronym of the collector; noun (name) in genitive case.

#### Distribution

**Figure 4.**
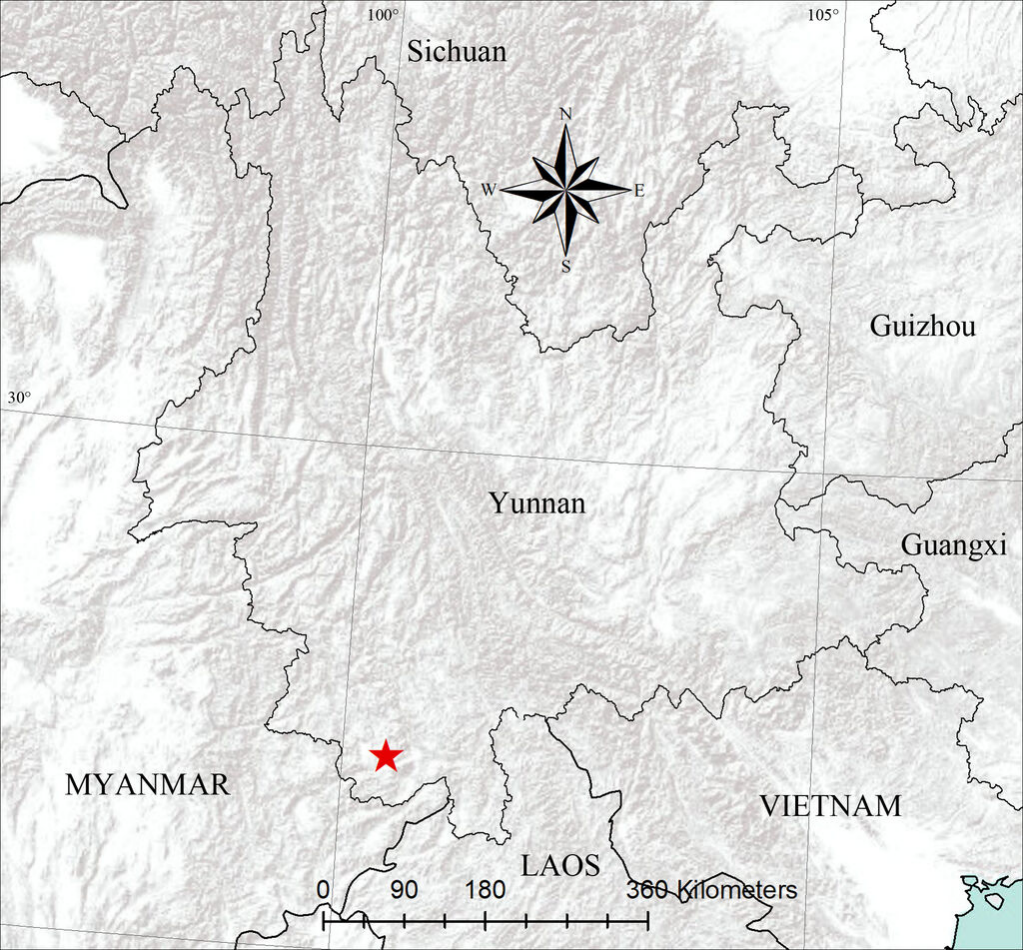
The distributional record of *Jerzegoqiuhangi* sp. nov. (red five-pointed star).

Known only from the type locality in Yunnan, China (Fig. 4).

## Supplementary Material

XML Treatment for Jerzego qiuhangi
